# 530. Compassionate Use of Skin Cell Suspension Autografts in Major Burns: A Single-center, US Experience

**DOI:** 10.1093/jbcr/irag033.227

**Published:** 2026-04-06

**Authors:** Hope Werenski, Jeffrey E Carter, Anju Saraswat, John Kevin Bailey, James H Holmes, IV

**Affiliations:** Wake Forest University School of Medicine, Winston-Salem, NC; University Medical Center Burn Center, Louisiana State University Health Sciences Center New Orleans, New Orleans, LA; Atrium Health Wake Forest Baptist, Winston-Salem, NC; Wake Forest University School of Medicine, Winston-Salem, NC; Atrium Health Wake Forest Baptist Burn Center, Winston-Salem, NC

## Abstract

**Introduction:**

Split-thickness autografts (STAG) remain the standard for closure of deep burns but are constrained by limited donor skin. At an expansion ratio of 80:1, non-cultured, autologous, skin cell suspension autografts (SCSA) allow for grafting with a reduction in donor skin. We report our single-center experience with SCSA as an adjunct to STAG in major burns.

**Methods:**

From 2014-2018, under an FDA-approved compassionate use (CU) protocol, 17 adults (≥40% TBSA) and 13 children (≥20% TBSA) were treated with SCSA at an ABA-verified burn center. SCSA was sprayed over widely meshed STAG and donor sites. Outcomes for treated adults were compared to controls from our Burn Registry, while treated children were compared to controls from the Burn Care Quality Platform (BCQP). Primary outcomes included length of stay (LOS) normalized to %TBSA and number of operations.

**Results:**

Seventeen adults (mean age 37 y, mean burn size 60% TBSA) [Table 1] and 13 children (mean age 3.5 y, mean burn size 39% TBSA) [Table 2] were treated under the CU SCSA protocol, with no deaths. Mean inpatient LOS per %TBSA was significantly reduced in adults treated with SCSA compared to controls (1.1 ± 0.6 d/%TBSA vs 1.9 ± 0.7 d/%TBSA, *p*=.001), while there was no observed difference for children. Notably, ~60% of our CU SCSA pediatric cases involved abuse/neglect or had other non-burn care issues requiring intensive social services that extended LOS beyond medical need. Finally, pediatric patients treated with SCSA underwent significantly fewer burn operations compared to controls (3 ± 2 vs 8 ± 8, *p*=.0001), while there was no observed difference for adults.

**Conclusions:**

In major burns, SCSA was feasible, safe, and associated with reduced LOS in adults and fewer operations in children. These findings support further study of SCSA as an adjunct to STAG in resource-intensive burn care.

**Applicability of Research to Practice:**

SCSA combined with STAG may reduce LOS and operative burden in major burns, thus improving outcomes and efficiency in modern burn care.

**Funding for the study:**

N/A.

